# Metabolic Perturbations Associated with an Exposure Mixture of Per- and Polyfluoroalkyl Substances in the Atlanta African American Maternal-Child Cohort

**DOI:** 10.1021/acs.est.3c04561

**Published:** 2023-10-19

**Authors:** Donghai Liang, Kaitlin R. Taibl, Anne L. Dunlop, Dana Boyd Barr, P. Barry Ryan, Todd Everson, Anke Huels, Youran Tan, Parinya Panuwet, Kurunthachalam Kannan, Carmen Marsit, Dean P. Jones, Stephanie M. Eick

**Affiliations:** † Gangarosa Department of Environmental Health, Rollins School of Public Health, Emory University, Atlanta, Georgia 30322, United States; ‡ Department of Gynecology and Obstetrics, School of Medicine, Emory University, Atlanta, Georgia 30322, United States; § Department of Epidemiology, Rollins School of Public Health, Emory University, Atlanta, Georgia 30322, United States; ∥ Department of Pediatrics, New York University School of Medicine, New York, New York 10016, United States; ⊥ Department of Environmental Medicine, New York University School of Medicine, New York, New York 10016, United States; # Division of Pulmonary, Allergy, and Critical Care Medicine, Department of Medicine, School of Medicine, Emory University, Atlanta, Georgia 30322, United States

**Keywords:** PFAS, high-resolution metabolomics, mixture analysis, quantile G-computation, environmental mixtures

## Abstract

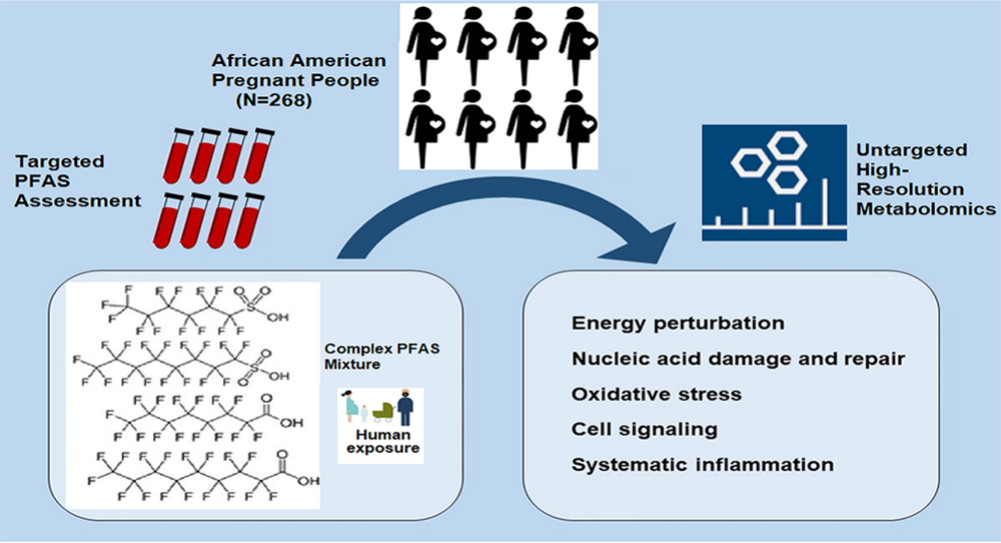

Prenatal exposure to single chemicals belonging to the per- and polyfluoroalkyl substances (PFAS) family is associated with biological perturbations in the mother, fetus, and placenta, plus adverse health outcomes. Despite our knowledge that humans are exposed to multiple PFAS, the potential joint effects of PFAS on the metabolome remain largely unknown. Here, we leveraged high-resolution metabolomics to identify metabolites and metabolic pathways perturbed by exposure to a PFAS mixture during pregnancy. Targeted assessment of perfluorooctanoic acid (PFOA), perfluorononanoic acid (PFNA), perfluorooctanesulfonic acid (PFOS), and perfluorohexanesulfonic acid (PFHxS), along with untargeted metabolomics profiling, were conducted on nonfasting serum samples collected from pregnant African Americans at 6–17 weeks gestation. We estimated the overall mixture effect and partial effects using quantile g-computation and single-chemical effects using linear regression. All models were adjusted for maternal age, education, parity, early pregnancy body mass index, substance use, and gestational weeks at sample collection. Our analytic sample included 268 participants and was socioeconomically diverse, with the majority receiving public health insurance (78%). We observed 13.3% of the detected metabolic features were associated with the PFAS mixture (*n* = 1705, *p* < 0.05), which was more than any of the single PFAS chemicals. There was a consistent association with metabolic pathways indicative of systemic inflammation and oxidative stress (e.g., glutathione, histidine, leukotriene, linoleic acid, prostaglandins, and vitamins A, C, D, and E metabolism) across all metabolome-wide association studies. Twenty-six metabolites were validated against authenticated compounds and associated with the PFAS mixture (*p* < 0.05). Based on quantile g-computation weights, PFNA contributed the most to the overall mixture effect for γ-aminobutyric acid (GABA), tyrosine, and uracil. In one of the first studies of its kind, we demonstrate the feasibility and utility of using methods designed for exposure mixtures in conjunction with metabolomics to assess the potential joint effects of multiple PFAS chemicals on the human metabolome. We identified more pronounced metabolic perturbations associated with the PFAS mixture than for single PFAS chemicals. Taken together, our findings illustrate the potential for integrating environmental mixture analyses and high-throughput metabolomics to elucidate the molecular mechanisms underlying human health.

## Abstract 2

Exposure to a PFAS mixture during early pregnancy may activate inflammatory and prooxidative pathways and dysregulate metabolites in the maternal metabolome.

## Introduction

A major and current public health concern is exposure to per- and polyfluoroalkyl substances (PFAS), a family of ≥9000 synthetic chemicals used in a wide range of industrial and commercial applications, including food packaging, stain-resistant furniture, and firefighting foam.^1^ PFAS are persistent in the environment and human exposure is ubiquitous, though variable across the United States (US), in part due to long biological half-lives and bioaccumulation.^2−4^ While concentrations of legacy PFAS, such as perfluorooctanesulfonic acid (PFOS) and perfluorooctanoic acid (PFOA), have gradually declined at the population level, replacement chemicals with similar structures continue to be introduced to the market and are associated with toxicity and exposure pathways.^3,5^

In recent years, a growing amount of epidemiological evidence has linked prenatal PFAS exposure to a range of adverse health outcomes, including adverse pregnancy and birth outcomes.^6−13^ Further, the maternal metabolome has been identified as a critical lens into the biomechanisms and biomarkers underlying exposure–outcome relationships. Metabolic perturbations that result from exposure to PFAS during pregnancy is associated with gestational diabetes, preterm birth, and fetal growth restriction.^14−16^ These metabolic perturbations are closely involved in the alteration of insulin sensitivity, lipid metabolism, and neuroendocrine signaling, among other pathways essential to maternal-placental-fetal health. Our work has also demonstrated that short-chain PFAS, such as perfluorohexanesulfonic acid (PFHxS), perturb the maternal metabolome through biosynthetic and bioenergetic pathways while legacy long-chain PFAS, such as PFOS and PFOA, tend to dysregulate signaling pathways.^15^ Despite awareness that individuals are exposed to multiple PFAS chemicals, the cumulative effects of PFAS on the human metabolome remain largely unknown due to the complexity of integrating high-dimensional data across statistical approaches.^17^ The assessment of biological responses to PFAS exposures and resulting health outcomes is further complicated by the lack of sensitive and specific biomarkers, interindividual heterogeneity in toxicokinetics, and involvement of numerous endogenous pathways.

Metabolomics is the systemic study of all metabolites associated with exogenous exposure and endogenous processes, and has emerged as an innovative and powerful analytical platform in environmental epidemiology.^18,19^ Recent investigations demonstrate the applicability of using metabolomics as a central platform to link human exposure with internal dose and biological response.^19−24^ Specifically, an increase in circulating concentrations of PFAS has been consistently associated with several endocrine disruption- and oxidative stress-related pathways.^15,16,25−27^ In one of our recent metabolome-wide association studies (MWAS), we also identified and verified several biomechanisms and biomarkers mediating the association between serum PFAS concentrations and fetal growth restriction.^15^ Moreover, in two separate analyses within the same pregnant population, we found an inverse association between prenatal exposure to PFAS, modeled as single chemicals and a mixture, with fetal growth measures.^28,29^

Despite these promising findings, methodological challenges remain in elucidating the potential biological responses and health effects associated with multiple PFAS chemicals, particularly for critical windows of exposure across the life course. The vast majority of metabolomics studies continue to focus on a single PFAS chemical at a time or the linear summation of PFAS concentrations, which prevents a deeper understanding about potential joint effects and neglects the high correlation of exposure within the PFAS family. The incorporation of chemical mixture models in MWAS is needed to perform comprehensive assessments of cumulative effects from multiple exposures. To address these knowledge gaps, we conducted a high-resolution metabolomics analysis with advanced environmental mixture methods via quantile g-computation to assess the single and potential joint effects of multiple PFAS on the maternal metabolome among 268 participants in the Atlanta African American Maternal-Child Cohort.

## Methods

### Study Population

The Atlanta African American Maternal-Child Cohort is an ongoing prospective birth cohort with participants recruited during prenatal visits from the Emory Healthcare and Grady Health systems in metropolitan Atlanta, Georgia. Participants were eligible for inclusion if they self-identified as a Black or African American female, were born in the United States, were between 18 and 40 years of age, were not pregnant with multiples, and had no chronic medical conditions or prescription medications. A detailed description of the recruitment and enrollment criteria has been previously published.^30,31^ For this analysis, we restricted the analytic sample to 268 participants, for whom information on PFAS exposure and untargeted high-resolution metabolomics profiling was available (Figure S1). All participants provided informed consent at enrollment, and this study was reviewed and approved by the Institutional Review Board of Emory University (approval reference number 68441).

### Data and Sample Collection

Maternal blood samples were collected during routine venipuncture between 6 and 17 weeks gestation and analyzed for targeted measurements of serum PFAS concentrations and untargeted high-resolution metabolomics profiling. Following collection, the samples were processed to obtain the serum, transported to the laboratory, and stored at −80 °C for future analyses, as previously described.^31^

Sociodemographic data was assessed using self-reported questionnaires and prenatal administrative record review and included maternal age at enrollment, maternal education, an income-to-poverty ratio, prenatal health insurance type, marital and relationship status, and substance use (alcohol, tobacco, and marijuana) during the previous month.

### PFAS Measurement

PFAS were quantified in serum samples at two laboratories within the Children’s Health Exposure Analysis Resource (CHEAR) program. The laboratories involved were the Wadsworth Center/New York University Laboratory Hub (Wadsworth/NYU) and the Laboratory of Exposure Assessment and Development for Environmental Research (LEADER) at Emory University. The samples were analyzed for PFHxS, PFOS, PFOA, and perfluorononanoic acid (PFNA) at both Wadsworth/NYU and LEADER. To ensure consistency and reliability of the results, the laboratories in CHEAR have engaged in activities to standardize measurements among them.^32^ Four PFAS (PFHxS, PFOS, PFOA, PFNA) were detected in >95% of maternal serum samples using high-performance liquid chromatography interfaced with tandem mass spectrometry (HPLC-MS/MS). The analytical methods used in both laboratories have been previously described and certified by the German External Quality Assessment Scheme twice annually. As previously reported, the results obtained from 11 overlapped samples showed good agreement between the laboratories, with Pearson correlation coefficients ranging from 0.88 to 0.93 and relative percent differences ranging from 0.12 to 20.2% (median 4.8%).^3^ PFAS concentrations below the limit of detections (LODs) were imputed with LOD/√2.^33^

### High-Resolution Metabolomics

We conducted untargeted high-resolution metabolomics profiling on nonfasting serum samples using a well-established protocol.^34−36^ As previously detailed in Chang et al.,^15^ two chromatography types were applied to the hydrophilic interaction liquid chromatography (HILIC) (Waters XBridge BEH Amide XP HILIC column; 2.1 × 50 mm^2^, 2.6 μm particle size) with positive electrospray ionization (ESI) and reversed-phase (C18) chromatography (Higgins Targa C18 2.1 × 50 mm2, 3 μm particle size) with negative ESI. Analyte separation for HILIC was performed using water, acetonitrile, and 2% formic acid mobile phases under the following gradient elution: initial 1.5 min period consisted of 22.5% water, 75% acetonitrile, and 2.5% formic acid, followed by a linear increase to 75% water, 22.5% acetonitrile, and 2.5% formic acid at 4 min, and a final hold of 1 min. Analyte separation for C18 was performed using water, acetonitrile, and 10 mM ammonium acetate mobile phases under the following gradient elution: the initial 1 min period consisted of 60% water, 35% acetonitrile, and 5% ammonium acetate followed by a linear increase to 0% water, 95% acetonitrile, and 5% ammonium acetate at 3 min and held for the remaining 2 min. For both types of chromatography, the mobile phase flow rate was 0.35 mL/min for the first minute and increased to 0.4 mL/min for the final 4 min. Although a gradient elution that starts at 60% aqueous condition in the C18 column might miss some metabolites, which could be separated between 100 and 60% aqueous, these metabolites are likely to be better detected in the HILIC column. Thus, the application of two chromatography types in this study can enhance the coverage of metabolic features for each sample. The void volume ends at approximately 15 s after injecting samples.

Liquid chromatography-high resolution mass spectrometry (LC-HRMS) was operated in full-scan mode at 120k resolution to cover a range of mass-to-charge ratio (*m*/*z*) from 85 to 1275, which includes features with a Level-1, -2, -3, or -4 confidence. Briefly, the evidence for chemical identifies confirmed with Level-1 confidence includes comparison to an authentic standard by mass spectrum and retention time; Level-2 confidence includes comparison of the mass spectrum to the library spectrum data where the spectrum structure match is unambiguous; Level-3 confidence includes tentative matches proposed with insufficient information for only one exact structure; and, Level-4 confidence includes only one reported unequivocal molecular formula.^19,37^ Two internal standards, which include pooled serum and standard reference material for human metabolites in plasma (NIST SRM 1950), were added at the beginning and end of each batch of 20 samples for quality control and standardization.^34,38^ The raw instrument files generated from the untargeted high-resolution metabolomics profiling were first converted to mzML format then metabolic features were extracted and aligned using apLCMS with modification of xMSanalyzer.^39,40^ This improved data quality control and reduced intra- and interbatch effects. Before statistical analyses, additional quality control measures were performed to optimize the data quality. Metabolic features detected in less than 15% of the samples, which had a coefficient of variation among technical replicates greater than 30%, and had a Pearson correlation coefficient less than 0.7 were excluded to filter out noise signals. The intensities of the remaining metabolic features were averaged across triplicates and log_2_-transformed to normalize the data for subsequent statistical analyses.

### Statistical Analysis

We conducted descriptive analyses for the serum PFAS concentrations, which involved the calculation of detection frequencies, geometric means (GMs), geometric standard deviations (GSDs), and distribution percentiles. Subsequently, we used two approaches to investigate the metabolic perturbations associated with four PFAS chemicals and their mixture. The metabolic features in all MWAS were analyzed without *a priori* knowledge of the actual chemical compound identities.

In our first approach, we used single-chemical linear regression models to evaluate the association between the intensity of each metabolic feature and the serum concentration of each PFAS chemical. In our second approach, we evaluated the potential joint effect using quantile g-computation, which estimates the association between the intensity of each metabolic feature and the serum concentration of the overall PFAS mixture. An important strength of quantile g-computation over single-chemical linear regression is that it better reflects real-world exposure patterns. Said differently, quantile g-computation estimates the effect of a simultaneous increase in all exposures within the PFAS mixture by 1-quartile. The quantile g-computation models also enabled insight into the mixture components that contributed the most and least to the cumulative effect by the weights for each PFAS chemical. Partial effects of single PFAS chemicals included in the mixture were estimated with positive weights, which were interpreted as synergism, and negative weights, which were interpreted as antagonism. Positive and negative weights sum to 1 in either direction and should not be directly compared. We selected quantile g-computation as the chemical mixture method due to ease of regression results comparison to linear regression and because it does not require directional homogeneity.^41^ Linear regression (“MASS” package) and quantile g-computation (“qgcomp” package) models were performed separately for each metabolic feature detected by the two different chromatography columns (HILIC and C18).

For both approaches, we retained maternal age, education, parity, early pregnancy BMI, history of substance use, and gestational age at sample collection as covariates in the models. These covariates were chosen based on a comprehensive literature review of potential confounding associations between exposures and outcomes in our study population (Figure S2).^3^ Previously, we have shown that pregnancy-related hemodynamics do not confound the association between prenatal PFAS exposure and fetal growth measures, so we did not adjust for these variables in any of the models.^28^ The Benjamini–Hochberg procedure was used to correct for multiple comparisons with the significance level set at 0.05 for corrected q-values, which helps to control the false discovery rate (FDR).^42^ FDR correction was performed for each MWAS. All analyses were conducted using R (version 4.1.0).

### Pathway Enrichment Analysis

To predict the pathway and biological functions of the significant features, we used *mummichog*, a statistical application that predicts the functional activity of metabolic pathways and networks without upfront chemical identification.^43^ Pathway enrichment analyses were conducted separately for PFNA, PFOA, PFOS, and PFHxS as well as the PFAS mixture containing the entire set of chemicals by two analytical columns. We visualized the enriched metabolic pathways associated with the single PFAS chemicals and their mixture with a bubble plot, where each bubble is shaded based on the strength of the associations in pathway enrichment analyses.

### Chemical Annotation and Confirmation

To reduce false positive discovery, we visually examined the extracted ion chromatographs (EICs) of each significant metabolic feature to differentiate true peak from noise (exhibiting clear Gaussian peak shapes and signal-to-noise ratios above 3:1). The features passing the examination were annotated and confirmed using the Metabolomics Standards Initiative criteria.^37^ Specifically, the features whose *m*/*z* (±10 ppm difference) and retention time (±30 s) matched the authentic compounds analyzed under identical experimental conditions were assigned with Level-1 confidence.

## Results

Our study population was composed of a socioeconomically diverse group of African American pregnant people (*N* = 268). Participant characteristics were representative of the overall cohort (Table 1). Over half of the participants reported use of tobacco, alcohol, or marijuana in the prior month (*N* = 146; 55%). The majority of participants had a high school education or less (*N* = 142; 53%) and Medicaid as their insurance (*N* = 210; 78%). At enrollment, the mean age of participants was 25.0 years (SD = 4.8), and the mean early pregnancy BMI was 29.0 kg/m^2^ ± 7.7 kg/m^2^. Four PFAS were detected in >95% of participants, with GMs of 0.98 (GSD = 1.98), 1.95 (GSD = 2.20), 0.63 (GSD = 2.42), and 0.24 ng/mL (GSD = 2.41) for PFHxS, PFOS, PFOA, and PFNA, respectively (Table 2). Pearson correlation coefficients between PFAS ranged from 0.37 to 0.76 (Figure S3).^15^

**Table 1:** Characteristics of Pregnant People in the Atlanta African American Maternal-Child Cohort, 2014–2020

characteristics	analytic sample	overall
*N*	268	525
Age (years)		
mean ± SD	25.0 ± 4.8	25.0 ± 4.9
missing	0	2 (0.4%)
Gestational age at sample collection (wks)		
mean ± SD	11.5 ± 2.2	11.0 ± 2.2
missing	0	2 (0.4%)
Sex of infant		
male	134 (50.0%)	252 (48.0%)
female	134 (50.0%)	264 (50.3%)
missing	0	9 (1.7%)
Parity		
mean ± SD	0.99 ± 1.1	0.94 ± 1.1
missing	0	2 (0.4%)
Prenatal body mass index (BMI; kg/m^2^)		
mean ± SD	29.0 ± 7.7	29.0 ± 7.8
missing	0	2 (0.4%)
Married or cohabiting		
yes	131 (48.9%)	249 (47.4%)
no	137 (51.1%)	274 (52.2%)
missing	0	2 (0.4%)
Medical insurance		
medicaid	210 (78.0%)	413 (78.7%)
private	58 (22.0%)	110 (21.0%)
missing	0	2 (0.4%)
Education level		
less than high school	38 (14.2%)	83 (15.8%)
high school	104 (38.8%)	202 (38.5%)
some college	81 (30.2%)	152 (29.0%)
college and above	45 (16.8%)	86 (16.4%)
missing	0	2 (0.4%)
Alcohol, marijuana, or tobacco use in the prior month		
yes	146 (54.5%)	292 (55.6%)
no	122 (45.5%)	231 (44.0%)
missing	0	2 (0.4%)

**Table 2:** Distribution of Serum PFAS Concentrations (ng/mL) in the Atlanta African American Maternal-Child Cohort, 2014–2020^a^

PFAS	ng/mL
PFOA	
detection rate^b^	97.0%
GM ± GSD	0.63 ± 2.42
P25–P75	0.47–1.09
max.	4.42
PFNA	
detection rate^b^	95.5%
GM ± GSD	0.24 ± 2.41
P25–P75	0.16–0.42
max.	2.27
PFOS	
detection rate^b^	98.5%
GM ± GSD	1.95 ± 2.20
P25–P75	1.42–3.12
max.	12.42
PFHxS	
detection rate^b^	95.9%
GM ± GSD	0.98 ± 1.98
P25–P75	0.75–1.52
max.	4.80

a Abbreviations: GM, geometric mean; GSD, geometric standard deviation; P25, 25th percentile; P50, 50th percentile; P75, 75th percentile; PFAS, per- and polyfluoroalkyl substances; PFOA, perfluorooctanoic acid; PFNA, perfluorononanoic acid; PFOS, perfluorooctanesulfonic acid; PFHxS, perfluorohexanesulfonic acid.

b The percentage of values above the limit of detection (LOD); values below the LOD were replaced by LOD/√2.

After QA/QC and data preprocessing, we extracted a total of 13,616 metabolic features from serum samples using the HILIC positive ESI column and 11,900 metabolic features using the C18 negative ESI column. We conducted a total of 10 MWAS to examine the associations between the four PFAS chemicals and their mixture using data from each of the chromatography columns. For the PFAS mixture MWAS, after FDR correction, one metabolic feature yielded a significant association in the HILIC column while 20 metabolic features yielded a significant association in the C18 column (*q* < 0.05). Because we found a limited number of significant features at either 5 or 20% FDR thresholds, the cutoff for significance was set as the unadjusted *p*-value < 0.05 to include a sufficient number of metabolic features in the pathway enrichment analyses.

**Figure 1:**
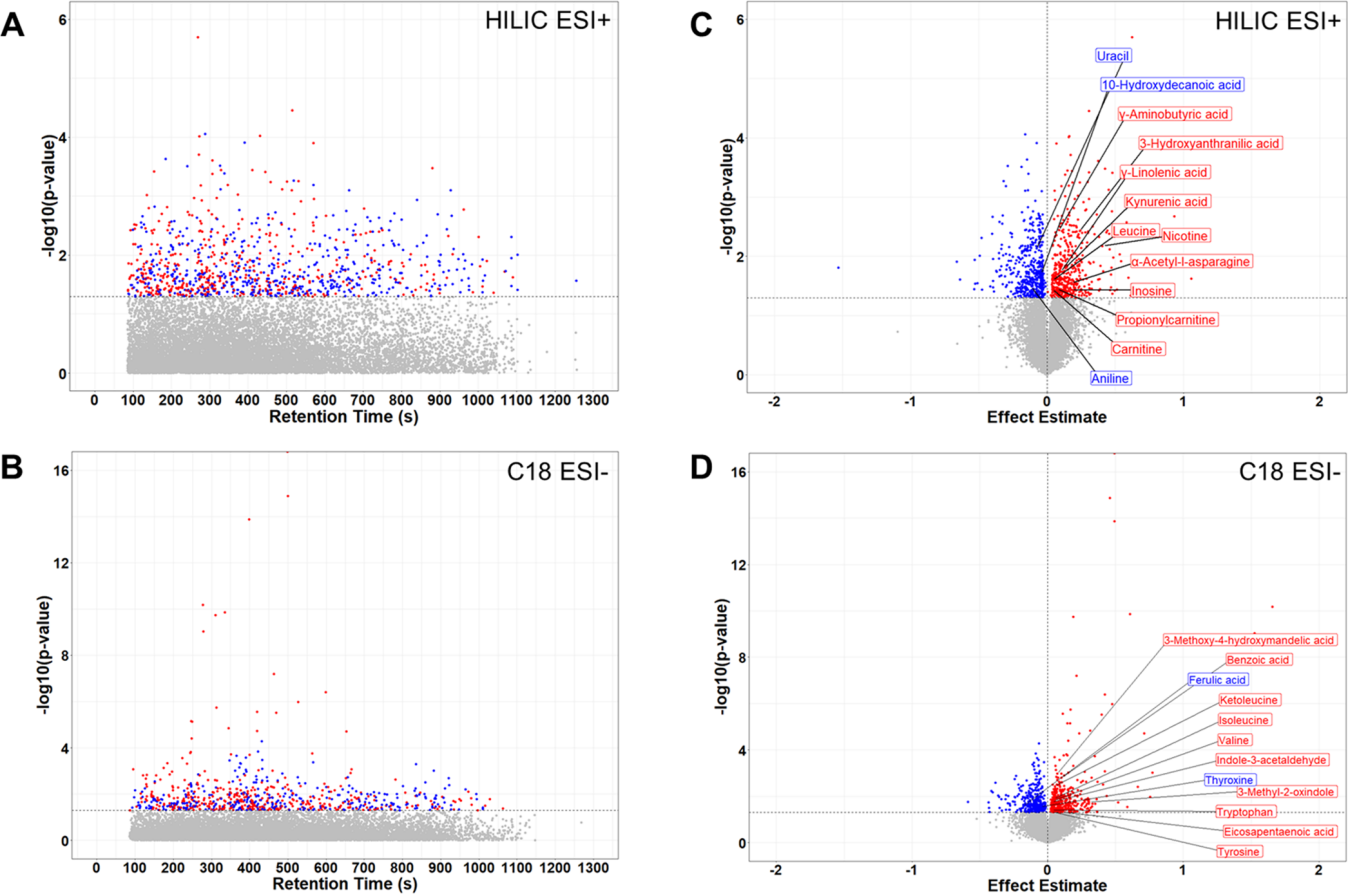
Manhattan plots (A, B) and Volcano plots (C, D) of metabolic features associated with the PFAS exposure mixture during early pregnancy. Note: Potential joint effects of PFAS mixture on maternal serum metabolome were examined by quantile g-computation. Red denotes a positive association with the PFAS mixture. Blue denotes a negative association with the PFAS mixture. Dashed lines refer to raw *p*-values; threshold is set to 0.05. Abbreviations: HILIC, hydrophilic interaction liquid chromatography column; C18, reversed-phase C18 column.

**Figure 2:**
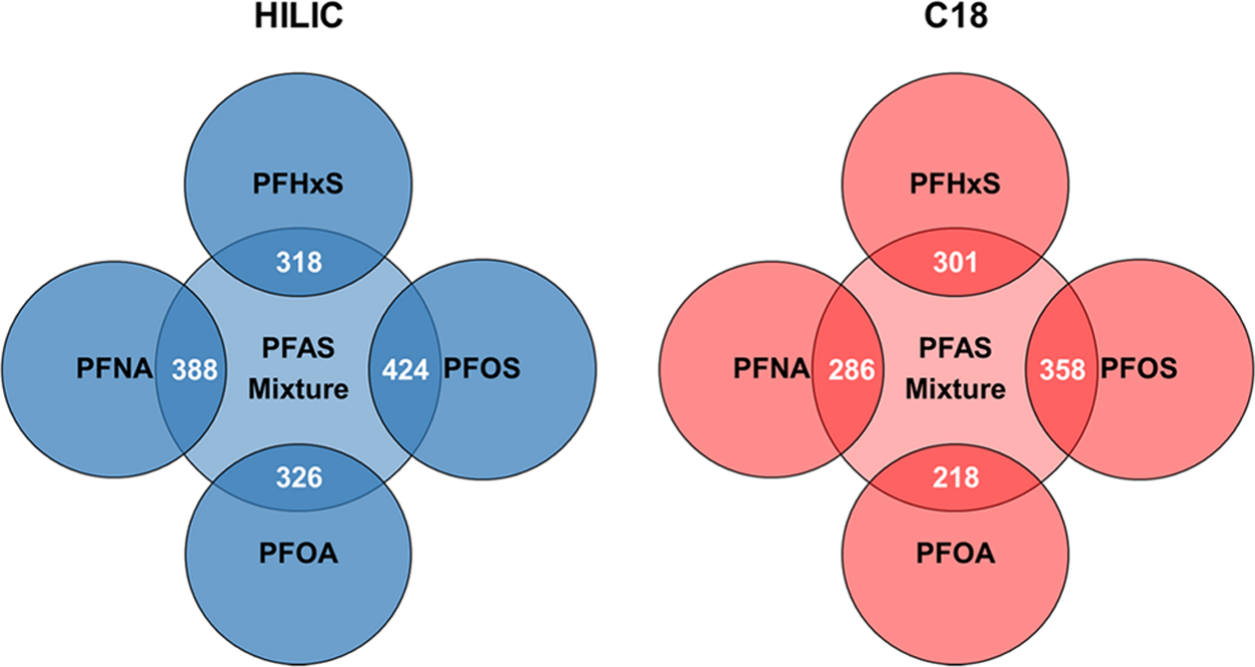
Venn diagrams of overlapping metabolic features associated with exposure to the PFAS mixture and individual PFAS chemicals during early pregnancy. Note: Potential joint effects of PFAS mixture on maternal serum metabolome were examined by quantile g-computation Abbreviations: HILIC, hydrophilic interaction liquid chromatography column; C18, reversed-phase C18 column.

Our single-chemical MWAS reflect the separate associations for each PFAS and are interpreted as follows: for every 1 ng/mL increase in natural log-transformed serum PFNA, PFOA, PFOS, and PFHxS at early pregnancy, there were 899, 736, 874, and 904 metabolic features significantly enriched in the maternal serum metabolome when analyzed by the HILIC column (*p* < 0.05). We identified a total of 531, 771, 664, and 674 significant metabolic features in the C18 column that were associated with PFOA, PFNA, PFOS, and PFHxS, respectively (*p* < 0.05). For the single PFAS chemicals, between 4.5 and 6.6% of the detected metabolic features were significantly enriched in the maternal serum metabolome (Table 3). Alternatively, our mixture MWAS reflect the potential joint effects of the PFAS mixture and are interpreted as follows: for a simultaneous increase in natural log-transformed serum PFOA, PFOS, PFHxS, and PFNA by 1-quartile, there were 971 and 734 metabolic features significantly enriched in the maternal serum metabolome when analyzed by the HILIC and C18 columns, respectively (*p* < 0.05). The HILIC column enrichment percentage was 7.1%, and the C18 column enrichment percentage was 6.2%. Across the 10 MWAS conducted, more metabolic features were significantly associated with the PFAS mixture (total *N* = 1705) than any of the single PFAS chemicals in both the HILIC and C18 columns together (Table 3 and Figure 1). Finally, the PFOS MWAS had the greatest number of overlapping metabolic features with the PFAS mixture MWAS (Figure 2).

**Table 3:** Metabolic Features Associated with Single PFAS Chemicals and Their Mixture during Early Pregnancy in the Atlanta African American Maternal-Child Cohort (*N* = 268), 2014–2020^a^

	HILIC (no. features = 13,616)						C18 (no. features = 11,900)					
	raw *p*-value < 0.05	% enriched	FDR *q*-value < 0.20	% enriched	FDR *q*-value < 0.05	% enriched	raw *p*-value < 0.05	% enriched	FDR *q*-value < 0.20	% enriched	FDR *q*-value < 0.05	% enriched
PFAS mixture^b^	971	7.1	1	0.01	1	0.01	734	6.2	37	0.31	20	0.17
PFOA	736	5.4	0	0	0	0	531	4.5	25	0.21	18	0.15
PFNA	899	6.6	0	0	0	0	771	6.5	35	0.29	21	0.18
PFOS	874	6.4	10	0.07	8	0.06	664	5.6	58	0.49	26	0.22
PFHxS	904	6.6	1	0.01	1	0.01	674	5.7	61	0.51	19	0.16

a Note: FDR indicates Benjamini–Hochberg procedure for false discovery rate correction of multiple comparisons. Models were adjusted for maternal age, education, parity, early pregnancy BMI, history of substance use, and gestational weeks at sample collection.

b Overall effect of the PFAS mixture was estimated using quantile g-computation.

**Figure 3:**
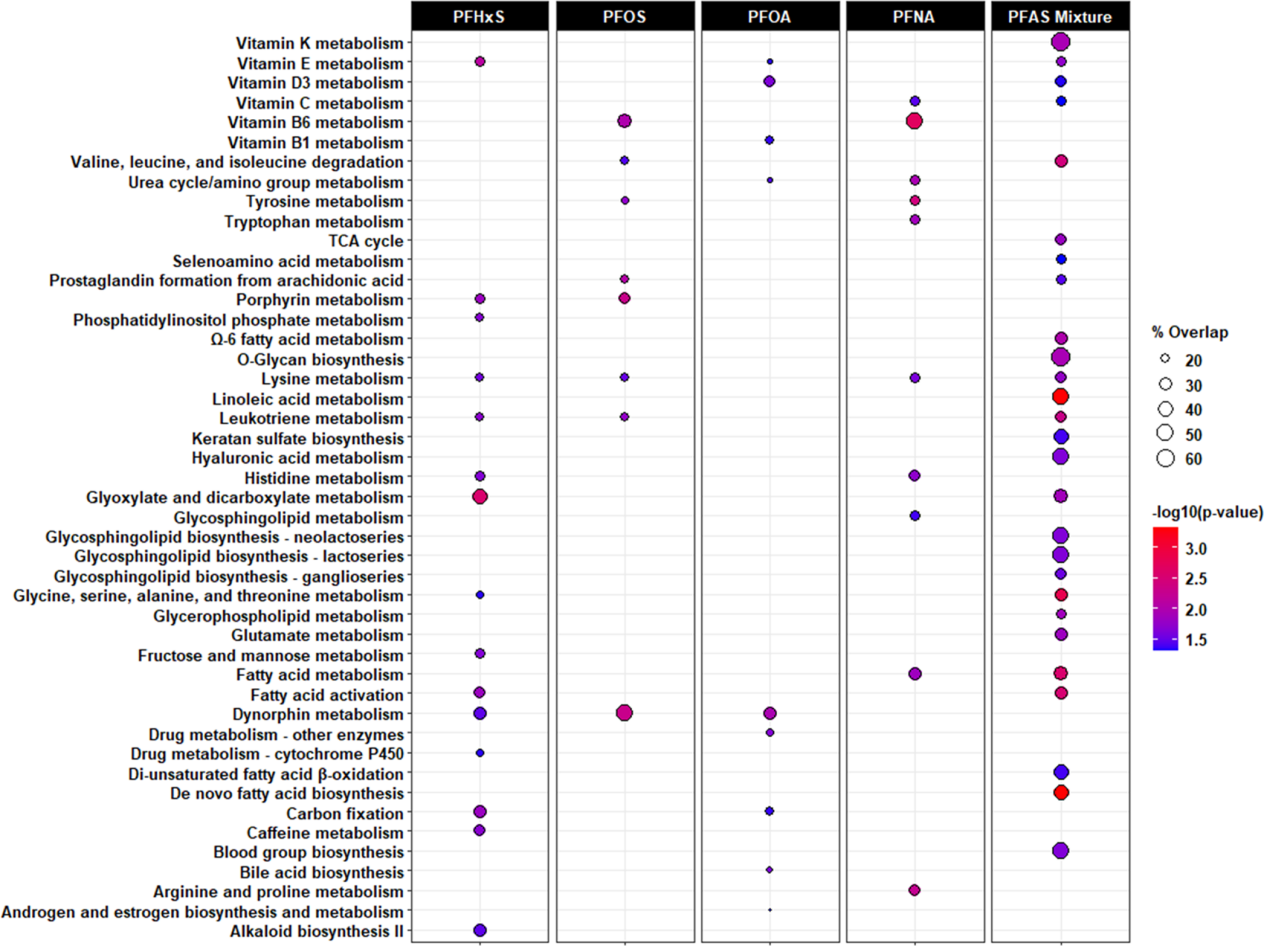
Metabolic pathways associated with exposure to individual PFAS and their mixture during early pregnancy in the C18 column. Note: Bubble color denotes the pathway significance level (−log_10_*p*-value). Bubble size denotes the percentage of significant metabolomic features (overlap size) versus the total number of metabolomic features within a pathway (pathway size). Only significant associations (*p* < 0.05) are indicated by bubbles in the plots. Only the following adducts were considered: M – H^[−]^, M + Cl^[−]^, M + ACN-H^[−]^, M + HCOO^[−]^, M(C13) – H^[−]^, M-H_2_O – H^[−]^, and M + Na – 2H^[−]^.

**Figure 4:**
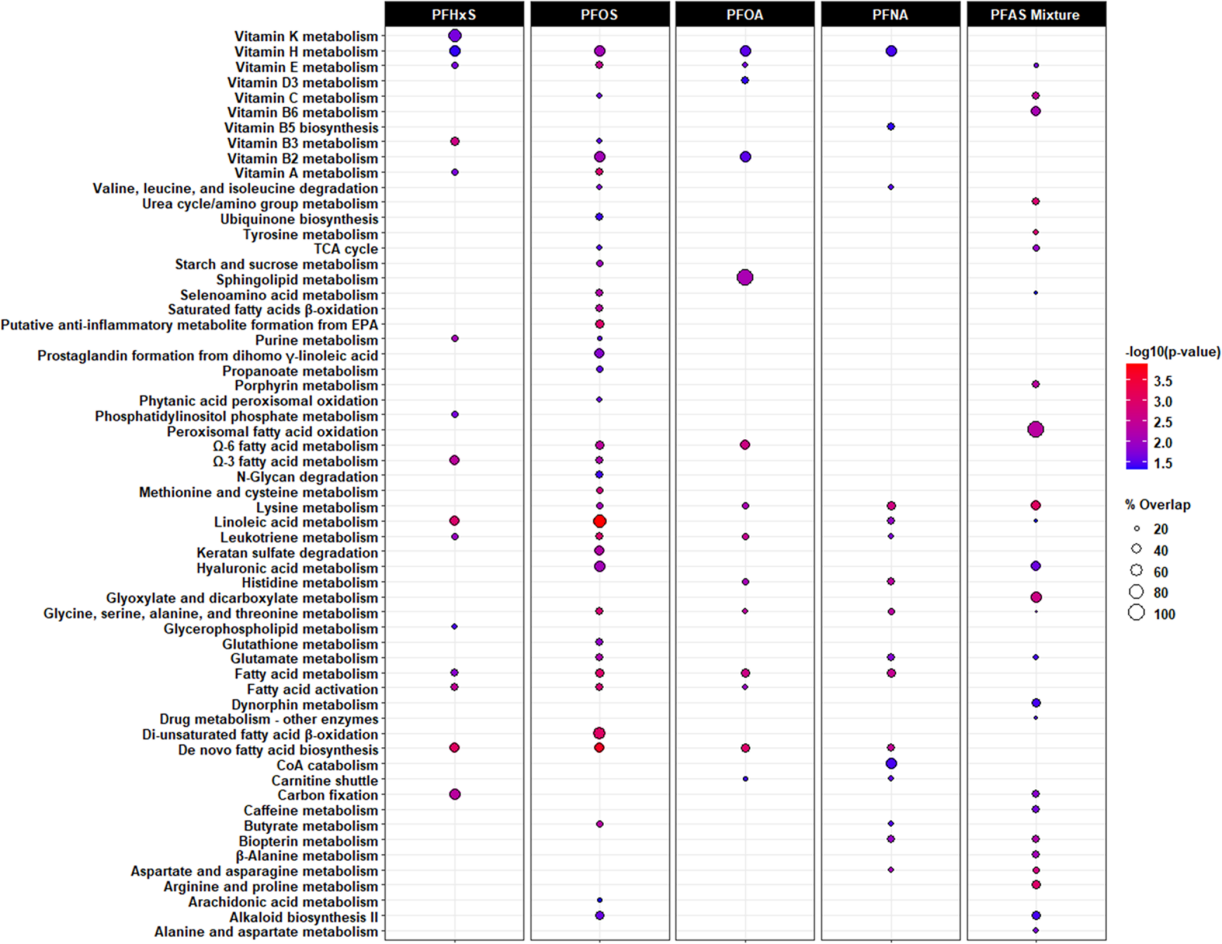
Metabolic pathways associated with exposure to individual PFAS and their mixture during early pregnancy in the HILIC column. Note: Bubble color denotes the pathway significance level (−log_10_*p*-value). Bubble size denotes the percentage of significant metabolomic features (overlap size) versus the total number of metabolomic features within a pathway (pathway size). Only significant associations (*p* < 0.05) are indicated by bubbles in the plots. Only the following adducts were considered: M^[1+]^, M + H^[1+]^, M – H_2_O + H^[1+]^, M + Na^[1+]^, M + K^[1+]^, M + 2H^[2+]^, and M(C13) + 2H^[2+]^.

Metabolic pathways associated with prenatal exposure to PFNA, PFOA, PFOS, PFHxS, or their mixture are shown in Figures 3 and 4, with detailed information provided in Tables S1 and S2. In the C18 column, we found the greatest number of pathways were enriched in the MWAS for PFNA and PFHxS, relative to those for PFOA and PFOS (Figure 3). Alternatively, in the HILIC column, there were 36 pathways enriched in the PFOS MWAS, out of the 74 identified across all MWAS (Figure 4). When comparing the pathways associated with any of the single PFAS chemicals to the PFAS mixture, we observed consistent metabolic perturbations involving systemic inflammation and oxidative stress, including metabolism of glutathione, histidine, leukotrienes, Ω-3 and Ω-6 fatty acids, prostaglandins, and vitamins A, C, D, and E. More interestingly, we found nine pathways exclusive to the PFAS mixture MWAS, which were not enriched in any of the single PFAS MWAS. For example, glycosphingolipid biosynthesis of neolacto-, lacto-, and ganglioseries in the C18 column as well as peroxisomal fatty acid oxidation and β-alanine metabolism in the HILIC column were associated with exposure to the PFAS mixture but not PFNA, PFOA, PFOS, or PFHxS. For the PFAS mixture MWAS, the percentage of metabolic features enriched in each pathway ranged from 19 to 100% among those measurable. Several pathways had a higher percentage of overlap and a lower *p*-value in the PFAS mixture MWAS than in any of the single PFAS chemicals MWAS, including lysine metabolism (HILIC column) and valine, leucine, and isoleucine degradation (C18 column).

Of the metabolic features significantly associated with the PFAS mixture, 26 metabolites were confirmed with Level-1 evidence using their chemical identity, *m*/*z*, and retention time (Table 4). During early pregnancy, the intensity of γ-aminobutyric acid (GABA) was higher by 0.09 (95% CI: 0.03, 0.15) in the maternal serum metabolome when the PFAS mixture was simultaneously increased by 1-quartile. In the HILIC column, 12 other metabolites, including γ-linolenic acid, carnitine, leucine, and uracil, were significantly associated with prenatal exposure to the PFAS mixture. Analysis of the maternal serum metabolome also revealed that a simultaneous one-quartile increase in the PFAS mixture was associated with a lower intensity of thyroxine (T4; ψ = −0.06; 95% CI: −0.11, −0.01). An additional 12 metabolites, including eicosapentaenoic acid (EPA), isoleucine, ketoleucine, tryptophan, tyrosine, and valine, were significantly associated with the PFAS mixture in the C18 column.

**Table 4:** Level-1 Confirmed Metabolites Associated with Exposure to the PFAS Mixture, Estimated Using Quantile g-Computation, during Early Pregnancy in the Atlanta African American Maternal-Child Cohort (*N* = 268), 2014–2020^a^

				overall mixture effect^b^		partial effects^c^			
metabolite	*m*/*z*	RT	column	ψ (95% CI)	*p*-value	PFOA	PFNA	PFOS	PFHxS
Amino acids									
α-acetyl-l-asparagine	175.0714	65.7	HILIC	0.18 (0.02, 0.34)	0.03	0.26	0.35	–1.00	0.39
3-hydroxyanthranilic acid	154.0499	35.4	HILIC	0.24 (0.08, 0.40)	0.004	–1.00	0.22	0.05	0.73
indole-3-acetaldehyde	158.0610	74.1	C18	0.03 (0.005, 0.05)	0.02	–0.18	0.65	–0.82	0.35
isoleucine	130.0874	23.9	C18	0.05 (0.01, 0.08)	0.01	–1.00	0.77	0.23	0.005
ketoleucine	129.0558	21.1	C18	0.05 (0.02, 0.09)	0.004	0.13	0.68	0.20	–1.00
leucine	132.1020	39.8	HILIC	0.04 (0.01, 0.08)	0.03	0.11	0.57	0.32	–1.00
tryptophan	203.0818	24.7	C18	0.03 (0.001, 0.05)	0.04	–0.72	0.67	–0.28	0.33
tyrosine	180.0665	23.3	C18	0.03 (0.001, 0.07)	0.048	–0.69	0.67	0.33	–0.31
valine	116.0718	24.7	C18	0.04 (0.01, 0.07)	0.02	–1.00	0.67	0.11	0.22
Fatty acids									
carnitine	162.1125	46.8	HILIC	0.04 (0.003, 0.07)	0.04	0.12	–1.00	0.70	0.17
eicosapentaenoic acid	301.2173	249.4	C18	0.12 (0.001, 0.23)	0.049	–1.00	0.14	0.43	0.43
γ-linolenic acid	279.2319	22.3	HILIC	0.10 (0.02, 0.19)	0.02	0.33	–1.00	0.45	0.22
propionylcarnitine	218.1386	32.5	HILIC	0.07 (0.01, 0.14)	0.04	0.15	0.33	0.41	0.11
Nucleic acids									
inosine	269.0883	43.3	HILIC	0.18 (0.01, 0.34)	0.04	0.13	–1.00	0.24	0.63
uracil	113.0347	39.0	HILIC	–0.08 (−0.13, −0.02)	0.01	0.75	–0.67	0.25	–0.33
Other endogenous metabolites									
γ-aminobutyric acid	104.0707	57.6	HILIC	0.09 (0.03, 0.15)	0.004	0.28	0.40	0.11	0.21
benzoic acid	121.0295	20.3	C18	0.10 (0.04, 0.17)	0.002	–0.82	–0.18	0.64	0.36
kynurenic acid	190.0498	37.2	HILIC	0.14 (0.03, 0.24)	0.02	–1.00	0.27	0.68	0.05
thyroxine	775.6796	46.6	C18	–0.06 (−0.11, −0.01)	0.03	–0.84	–0.03	–0.05	–0.07
Exogenous metabolites									
aniline	94.0653	39.1	HILIC	–0.11 (−0.22, −0.01)	0.03	–0.19	–0.28	1.00	–0.53
ferulic acid	195.0661	23.7	C18	–0.18 (−0.34, −0.02)	0.03	–0.06	1.00	–0.25	–0.69
10-hydroxydecanoic acid	189.1483	22.7	HILIC	–0.04 (−0.07, −0.01)	0.02	1.00	–0.27	–0.26	–0.48
3-methoxy-4-hydroxymandelic acid	197.0434	22.3	C18	0.07 (0.03, 0.11)	0.001	0.43	0.55	0.02	–1.00
3-methyl-2-oxindole	162.0559	28.8	C18	0.30 (0.05, 0.55)	0.02	0.86	0.05	–1.00	0.10
nicotine	163.1230	34.5	HILIC	0.41 (0.12, 0.70)	0.01	0.04	0.38	0.14	0.44
oxovaleric acid	115.0402	22.9	C18	0.05 (0.01, 0.08)	0.01	0.39	0.43	–1.00	0.18

a Note: Models were adjusted for maternal age, education, parity, early pregnancy BMI, history of substance use, and gestational weeks at sample collection.

b Overall effect of the PFAS mixture on the maternal serum metabolome examined by quantile g-computation. ψ estimates are interpreted as the overall mixture effect on the intensity of a maternal metabolite for a simultaneous increase in each PFAS chemical by 1-quartile.

c Direction (±) of weights indicate positive (+) or negative (−) effects. The magnitude (−1 to 1) of weights indicates relative importance to the overall mixture effect in either direction and should not be directly compared.

We observed several patterns of synergism and antagonism between PFAS chemicals in the exposure mixture associated with Level-1 confirmed metabolites (Table 4). PFNA exhibited antagonistic effects (i.e., negative quantile g-computation weights) on metabolites related to nucleic acids and synergistic effects (i.e., positive quantile g-computation weights) on metabolites related to amino acids. Finally, the overall mixture effect on the fatty acid and lipid metabolite intensities was predominantly driven by PFOS, based on the direction and magnitude of quantile g-computation weights.

## Discussion

In one of the first studies of its kind, we demonstrate the feasibility and utility of using statistical methods designed for exposure mixtures in combination with high-resolution metabolomics to investigate the cumulative effects of prenatal PFAS exposure on the maternal metabolome. Overall, we found more pronounced metabolic perturbations associated with the PFAS mixture than with any of the single PFAS chemicals. Our findings indicate that simultaneous exposure to PFNA, PFOA, PFOS, and PFHxS during early pregnancy is associated with inflammatory and prooxidative pathways and metabolites. Specifically, the biological perturbations suggested systemic inflammation, endocrine disruption, nucleic acid damage, and redox dyshomeostasis, which may guide future public health and clinical interventions. The patterns observed across all of the MWAS also illustrate the consistency of this method, which may be leveraged in future studies to examine the joint impact of environmental exposures on the human metabolome in association with health outcomes.

The majority of population-based metabolomics studies have assessed the effect of a single environmental chemical. However, these models do not accurately reflect how human exposures occur in reality, as more than 350,000 chemicals and their mixtures exist in commerce and cumulative exposures may produce additive or synergistic effects.^44^ In the present study, we utilized quantile g-computation to assess the cumulative and potential joint effects of a PFAS mixture, thereby providing an important proof-of-concept for future work. The serum concentrations of most PFAS in our study population were comparable to participants with the same race, age, and sex in US NHANES and pregnant people who identify as a minority racial/ethnic group in other prospective cohorts during the same time frame; a notable exception is PFHxS, which is significantly higher in the Atlanta African American Maternal-Child Cohort.^3,16^ Furthermore, we observed that more metabolic features were associated with the PFAS mixture containing PFNA, PFOA, PFOS, and PFHxS relative to when we assessed their individual effects in single-chemical models across both analytical columns, which has important implications for precision environmental health. To our knowledge, this is the first application of quantile g-computation in high-throughput -omics analyses examining the impact of environmental mixtures on the human metabolome.^14,21,22,45^ This approach allows for continuous and categorical outcomes, which can be easily incorporated into the meet-in-the-middle approach and high-dimensional mediational analysis in the future to examine how the metabolic perturbations mediate the associations between mixtures of environmental pollutants and complex adverse health outcomes.^46^

In previous work, we reported on the maternal and fetal metabolomic associations with prenatal exposure to single PFAS chemicals and adverse birth outcomes.^15,16^ The present analysis builds on this work by examining the effects of a PFAS mixture. Similar to our approach, a 2023 study also examined the impact of a PFAS mixture on the plasma metabolome of adolescents and young adults enrolled in the Study of Latino Adolescents at Risk (SOLAR) and Southern California Children’s Health Study (CHS).^17^ Across our study and the study conducted within SOLAR and CHS, amino acids and fatty acids critical to energy production and endocrine signaling for healthy growth and development in early life were consistently perturbed. In addition, we have shown in two separate study populations that pregnant people exposed to higher levels of PFNA, PFOA, PFOS, PFHxS, perfluorodecanoic acid (PFDA), and perfluoroundecanoic acid (PFUNDA), and their mixture, are associated with elevated oxidative stress.^47^ Our prior findings align with those presented in the current study. A diverse suite of inflammatory (e.g., histidine metabolism, prostaglandin formation, leukotriene metabolism, Ω-6 fatty acid metabolism), proteinogenic [e.g., branched-chain amino acid (BCAA) metabolism, tyrosine metabolism, vitamin K metabolism], and redox or otherwise bioenergetic-related (e.g., CoA biosynthesis and degradation, glutathione metabolism, fatty acid β-oxidation, TCA cycle, glycerophospholipid biosynthesis and metabolism, carnitine shuttle) pathways were also enriched in our PFAS mixture MWAS. A similar set of maternal metabolites were negatively associated with the PFAS mixture, including T4 and uracil, which were mainly driven by PFOA and PFNA, respectively. In contrast, PFNA had a positive partial effect for all amino acid metabolites, as well as GABA, with the greatest contribution to the overall mixture effect for 11 of the 26 confirmed metabolites. This observation may reflect the wide usage and longstanding production of PFNA, a long-chain PFAS, that only recently began to phase out in the US.

Often referred to as pyridoxine, vitamin B6 is essential to the nervous system and functions as a cofactor for the biosynthesis of neurotransmitters.^48^ We found that vitamin B6 metabolism was associated with prenatal exposure to PFOS and PFNA in single-chemical MWAS, as well as the PFAS mixture. Consistent with pathway enrichment analysis, the effect of the PFAS mixture on GABA was primarily driven by PFNA, as evidenced by PFNA having the largest partial effect. The ability for *in utero* exposure to PFAS to cross the blood–brain barrier, particularly long-chain chemicals like PFNA, and bioaccumulate in lipid-rich tissues, such as the brain, have raised concerns about neurotoxic health effects even before birth.^49,50^ However, there are mixed reports of a link between prenatal PFAS exposures and altered neurodevelopment or neurotransmitters in laboratory and population-based settings.^51−54^ Our findings provide a potential mechanism and marker, vitamin B6 metabolism and GABA—to investigate in follow-up investigations of PFAS mixtures.

Pathways enriched in the PFAS mixture of MWAS with the greatest size and strength were related to fatty acid biosynthesis, activation, metabolism, and oxidation as well as eicosanoid metabolism. Many of these pathways were also enriched in the single-chemical PFOS MWAS. Furthermore, the PFAS mixture had a positive cumulative effect on EPA (an Ω-3 fatty acid), γ-linolenic acid (an Ω-6 fatty acid), and carnitine, which shuttles long-chain fatty acids to the mitochondrion for β-oxidation. For each of these, the effect of the PFAS mixture was driven by PFOS. These findings are supported by prior work that has shown exposures to PFOS and other PFAS chemicals interfere with lipid homeostasis in the mother, placenta, and fetus.^55^ For example, lipid-derived biomarkers of systemic inflammation, oxidative stress, and hormone function are altered by simultaneous exposure to multiple PFAS chemicals during the perinatal period.^47,56,57^ The results we present align with previous epidemiologic work and contribute new information about how fatty acid and lipid metabolism are jointly affected by the four most commonly detected and highly concentrated PFAS in Americans.

An important strength of our study is the use of comprehensive, high-resolution metabolomics methods to assess global metabolism profiles and investigate broad metabolic changes. Additionally, we utilized quantile g-computation to characterize the cumulative and potential joint effects of a PFAS mixture on the maternal metabolome, which we compared to patterns observed with single PFAS chemicals. Second, our study population was exclusive to African Americans, who are often exposed to higher levels of environmental hazards and experience a disproportionate burden of adverse pregnancy outcomes. Nonetheless, we acknowledge that this limits our external generalizability to other racial and ethnic groups, as well as nonpregnant populations; although, the consistent perturbations identified in our study are similar to metabolic pathways previously reported in independent studies and other populations.^25,26,58,59^

Our study had several other limitations. The PFAS concentrations and metabolic features were both assessed in serum samples obtained between 6 and 17 weeks gestation. In this sense, our study is cross-sectional. Nonfasting status may also introduce measurement variation. However, we believe any measurement error is minimal, as we used pool standards and internal references in the metabolic profiling plus followed a comprehensive metabolomics workflow to minimize the potential impact of nonfasting status, as successfully demonstrated in previous studies.^34,60^ Additionally, we did not consider dietary predictors, such as fish consumption or drinking water, which may be a source of unmeasured confounding, and cannot rule out the possibility that participants had an unknown, undiagnosed medical condition or took medications or supplements during pregnancy, which may have influenced our results. At the time of analysis, we did not have information on previous breastfeeding practices either, an established determinant of maternal PFAS body burden. Further, our results may be subject to recall bias for specific confounders (e.g., substance use), where participants may be more likely to under-report usage. Lastly, given the purpose of this proof-of-concept analysis is to demonstrate the feasibility and utility of applying mixture analysis in metabolomics application, we used unadjusted *p*-values for the pathway enrichment analysis due to insufficient power, which is common in environmental metabolomics studies.^19^ Despite the use of a less conservative significance cutoff, we identified pathways and metabolites similar to other PFAS MWAS that corrected for multiple comparisons.^16,61^

## Implications

This study presents a novel approach to examine the combined effects of multiple PFAS exposures on the maternal metabolome using environmental chemical mixture methods in conjunction with high-resolution metabolomics. Our findings suggest that exposure to multiple PFAS during early pregnancy may activate inflammatory and prooxidative pathways and metabolites in the maternal metabolome. Our study serves as a proof of concept of how high-throughput omics and environmental molecular epidemiology can be integrated, which is an important step toward a precision medicine model in public health and translational science.

## Supporting Information Available

Metabolic pathways significantly associated with single PFAS chemicals and their exposure mixture in the C18 negative ESI column (Table S1); metabolic pathways significantly associated with single PFAS chemicals and their exposure mixture in the HILIC positive ESI column (Table S2); flowchart of participants included in the analytic sample from the Atlanta African American Maternal-Child Cohort, 2014–2020 (Figure S1); directed acyclic graph (DAG) showing the hypothesized relationships among maternal PFAS exposure, maternal metabolome, and maternal characteristics (Figure S2); correlation matrix of serum PFAS concentrations in 268 pregnant people in the Atlanta African American Maternal-Child Cohort, 2014–2020 (Figure S3) (PDF)
