# Supplementary material for: Metabolic Perturbations Associated with an Exposure Mixture of Per- and Polyfluoroalkyl Substances in the Atlanta African American Maternal-Child Cohort
Source: Environ Sci Technol. 2023 Oct 19;57(43):16206–18. doi: 10.1021/acs.est.3c04561 (PMC10620983; doi:10.1021/acs.est.3c04561)
Supplement: Supplementary file 1 — es3c04561_si_001.pdf [file es3c04561_si_001.pdf]

<u>**Supporting Information**</u>

Metabolic Perturbations Associated with an Exposure Mixture of Per- and Polyfluoroalkyl Substances in the Atlanta African American Maternal-Child Cohort

Authors:
Donghai Liang[1^], Kaitlin R. Taibl[1^], Anne L. Dunlop[2], Dana Boyd Barr[1], P. Barry Ryan[1], Todd Everson[1], Anke Huels[3,1], Youran Tan[1], Parinya Panuwet[1], Kurunthachalam Kannan[4,5], Carmen Marsit[1], Dean P. Jones[6], Stephanie M. Eick[1]

Affiliations:
[1]Gangarosa Department of Environmental Health, Rollins School of Public Health, Emory University, Atlanta, GA, 30322, USA
[2]Department of Gynecology and Obstetrics, School of Medicine, Emory University, Atlanta, GA, 30322, USA
[3]Department of Epidemiology, Rollins School of Public Health, Emory University, Atlanta, GA, 30322, USA
[4]Department of Pediatrics, New York University School of Medicine, New York, NY, 10016, USA
[5]Department of Environmental Medicine, New York University School of Medicine, New York, NY, 10016, USA
[6]Division of Pulmonary, Allergy, and Critical Care Medicine, Department of Medicine, School of Medicine, Emory University, Atlanta, GA, 30322, USA

^ Authors contributed equally

Corresponding Authors:
Donghai Liang, PhD, MPH
Assistant Professor, Gangarosa Department of Environmental Health
Emory University Rollins School of Public Health
1518 Clifton Rd, Rm 7021
Atlanta, GA 30322, USA
donghai.liang@emory.edu

Stephanie M. Eick, PhD, MPH
Assistant Professor, Gangarosa Department of Environmental Health
Emory University Rollins School of Public Health
1518 Clifton Rd, Rm 7027
Atlanta, GA 30322, USA
stephanie.marie.eick@emory.edu

Number of Pages: 8
Number of Figures: 3
Number of Tables: 2

Figure S1. Flowchart of participants included in the analytic sample from the Atlanta African American Maternal-Child Cohort, 2014 – 2020.

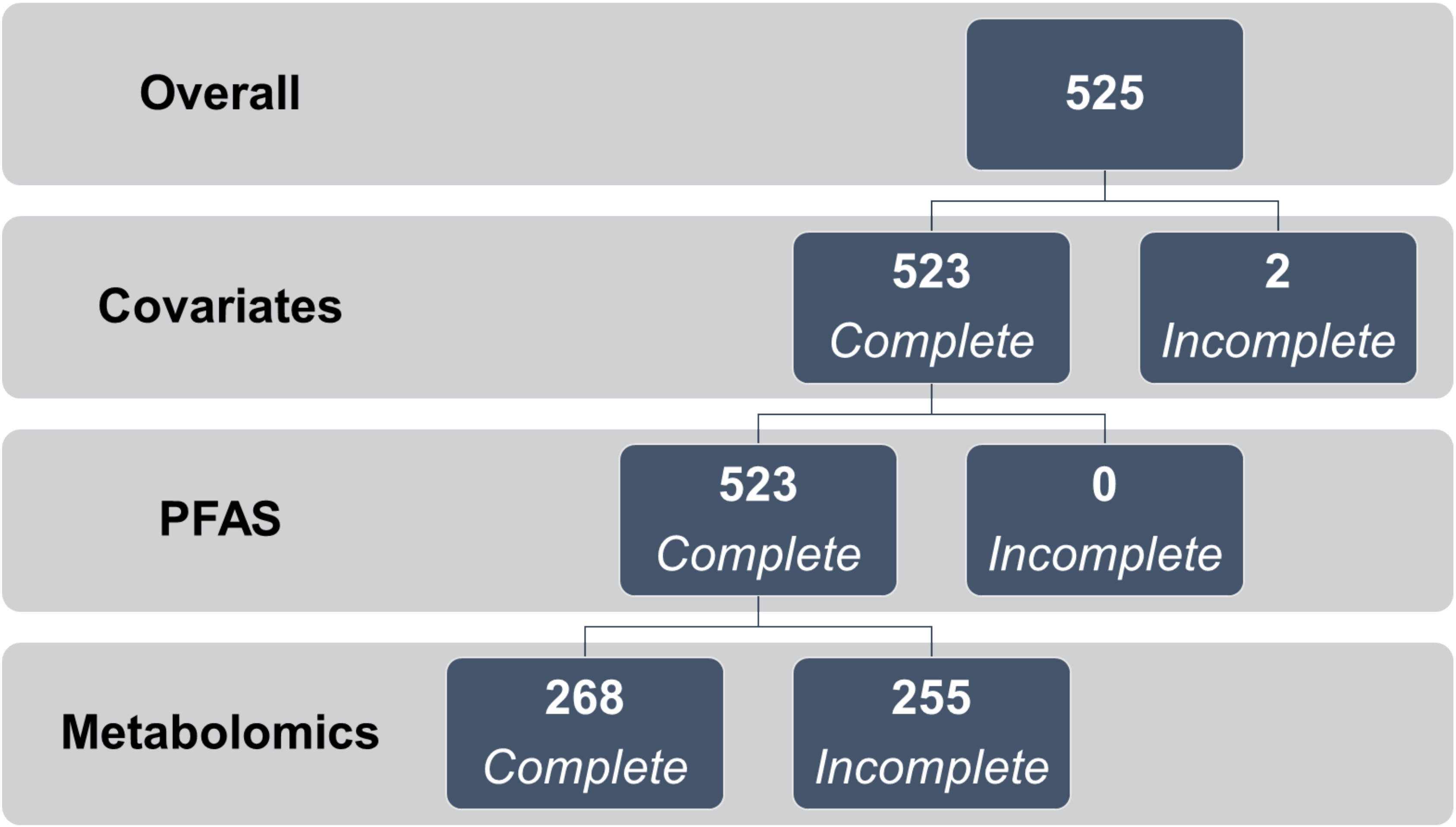

Figure S2. Directed acyclic graph (DAG) showing the hypothesized relationships among maternal PFAS exposure, maternal metabolome, and maternal characteristics.

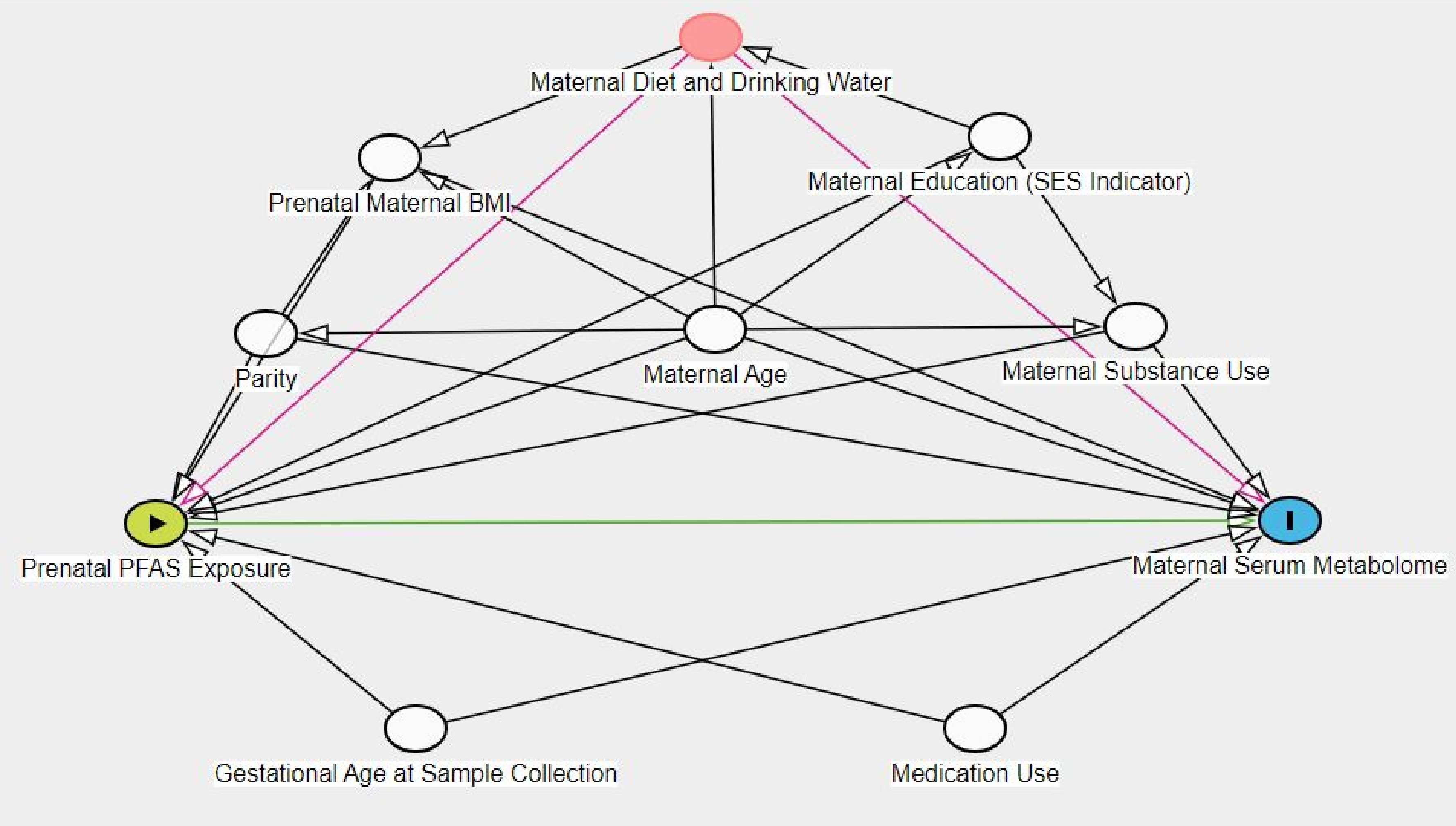

Figure S3. Correlation matrix of serum PFAS concentrations in 268 pregnant people in the Atlanta African American Maternal-Child Cohort, 2014 – 2020.

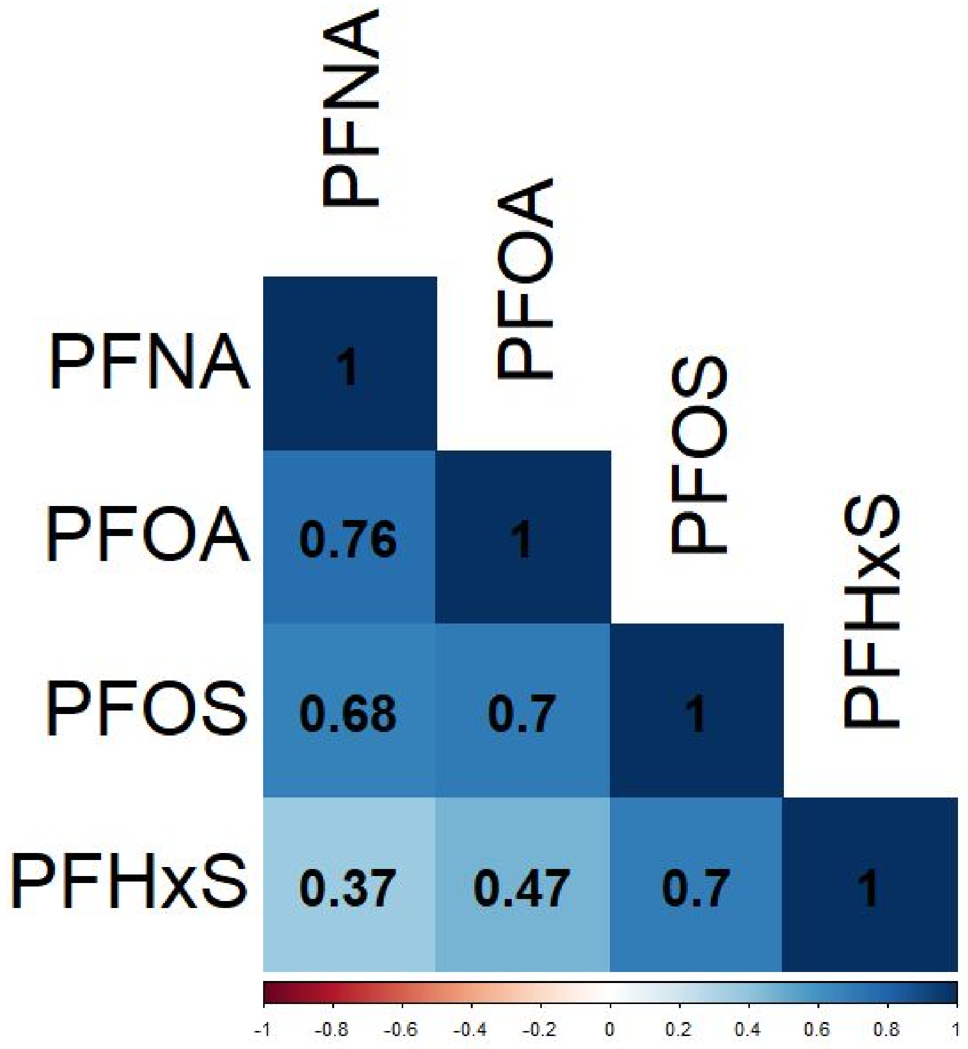

Table S1. Metabolic pathways significantly associated with single PFAS chemicals and their exposure mixture in the C18 negative ESI column.

Pathway	C18 ESI- Column														
	PFHxS MWAS			PFOS MWAS			PFOA MWAS			PFNA MWAS			PFAS Mixture MWAS		
	Overlap	Pathway	p-value	Overlap	Pathway	p-value	Overlap	Pathway	p-value	Overlap	Pathway	p-value	Overlap	Pathway	p-value
Alanine and aspartate metabolism	-	-	-	-	-	-	-	-	-	-	-	-	-	-	-
Alkaloid biosynthesis II	2	6	0.033	-	-	-	-	-	-	-	-	-	-	-	-
Androgen and estrogen biosynthesis and metabolism	-	-	-	-	-	-	8	67	0.047	-	-	-	-	-	-
Arachidonic acid metabolism	-	-	-	-	-	-	-	-	-	-	-	-	-	-	-
Arginine and proline metabolism	-	-	-	-	-	-	-	-	-	9	34	0.006	-	-	-
Aspartate and asparagine metabolism	-	-	-	-	-	-	-	-	-	-	-	-	-	-	-
β-Alanine metabolism	-	-	-	-	-	-	-	-	-	-	-	-	-	-	-
Bile acid biosynthesis	-	-	-	-	-	-	6	44	0.023	-	-	-	-	-	-
Biopterin metabolism	-	-	-	-	-	-	-	-	-	-	-	-	-	-	-
Blood group biosynthesis	-	-	-	-	-	-	-	-	-	-	-	-	2	4	0.023
Butyrate metabolism	-	-	-	-	-	-	-	-	-	-	-	-	-	-	-
Caffeine metabolism	3	11	0.020	-	-	-	-	-	-	-	-	-	-	-	-
Carbon fixation	3	10	0.015	-	-	-	2	10	0.043	-	-	-	-	-	-
Carnitine shuttle	-	-	-	-	-	-	-	-	-	-	-	-	-	-	-
CoA catabolism	-	-	-	-	-	-	-	-	-	-	-	-	-	-	-
De novo fatty acid biosynthesis	-	-	-	-	-	-	-	-	-	-	-	-	14	31	0.0005
Di-unsaturated fatty acid β-oxidation	-	-	-	-	-	-	-	-	-	-	-	-	2	5	0.040
Drug metabolism - cytochrome P450	8	48	0.045	-	-	-	-	-	-	-	-	-	-	-	-
Drug metabolism - other enzymes	-	-	-	-	-	-	4	26	0.022	-	-	-	-	-	-
Dynorphin metabolism	2	6	0.033	3	6	0.005	2	6	0.011	-	-	-	-	-	-
Fatty acid activation	4	16	0.015	-	-	-	-	-	-	-	-	-	9	30	0.003
Fatty acid metabolism	-	-	-	-	-	-	-	-	-	4	13	0.014	6	17	0.003
Fructose and mannose metabolism	5	24	0.022	-	-	-	-	-	-	-	-	-	-	-	-
Glutamate metabolism	-	-	-	-	-	-	-	-	-	-	-	-	4	13	0.014
Glutathione metabolism	-	-	-	-	-	-	-	-	-	-	-	-	-	-	-
Glycerophospholipid metabolism	-	-	-	-	-	-	-	-	-	-	-	-	11	46	0.013
Glycine, serine, alanine, and threonine metabolism	8	48	0.045	-	-	-	-	-	-	-	-	-	14	47	0.001
Glycosphingolipid biosynthesis - ganglioseries	-	-	-	-	-	-	-	-	-	-	-	-	4	15	0.029
Glycosphingolipid biosynthesis - lactoseries	-	-	-	-	-	-	-	-	-	-	-	-	2	4	0.023
Glycosphingolipid biosynthesis - neolactoseries	-	-	-	-	-	-	-	-	-	-	-	-	2	4	0.023
Glycosphingolipid metabolism	-	-	-	-	-	-	-	-	-	6	27	0.040	-	-	-
Glyoxylate and dicarboxylate metabolism	4	9	0.003	-	-	-	-	-	-	-	-	-	3	8	0.013
Histidine metabolism	5	24	0.022	-	-	-	-	-	-	6	24	0.018	-	-	-
Hyaluronic acid metabolism	-	-	-	-	-	-	-	-	-	-	-	-	2	4	0.023
Keratan sulfate biosynthesis	-	-	-	-	-	-	-	-	-	-	-	-	2	5	0.040
Keratan sulfate degradation	-	-	-	-	-	-	-	-	-	-	-	-	-	-	-
Leukotriene metabolism	9	48	0.019	9	48	0.017	-	-	-	-	-	-	13	50	0.005
Linoleic acid metabolism	-	-	-	-	-	-	-	-	-	-	-	-	11	22	0.0005
Lysine metabolism	5	25	0.026	5	25	0.030	-	-	-	6	25	0.024	7	28	0.017
Methionine and cysteine metabolism	-	-	-	-	-	-	-	-	-	-	-	-	-	-	-
N-Glycan degradation	-	-	-	-	-	-	-	-	-	-	-	-	-	-	-

O-Glycan biosynthesis	-	-	-	-	-	-	-	-	-	-	-	-	2	3	0.011
Ω-3 fatty acid metabolism	-	-	-	-	-	-	-	-	-	-	-	-	-	-	-
Ω-6 fatty acid metabolism	-	-	-	-	-	-	-	-	-	-	-	-	4	12	0.010
Peroxisomal fatty acid oxidation	-	-	-	-	-	-	-	-	-	-	-	-	-	-	-
Phosphatidylinositol phosphate metabolism	6	30	0.021	-	-	-	-	-	-	-	-	-	-	-	-
Phytanic acid peroxisomal oxidation	-	-	-	-	-	-	-	-	-	-	-	-	-	-	-
Porphyrin metabolism	5	22	0.015	6	22	0.005	-	-	-	-	-	-	-	-	-
Propanoate metabolism	-	-	-	-	-	-	-	-	-	-	-	-	-	-	-
Prostaglandin formation from arachidonic acid	-	-	-	12	62	0.009	-	-	-	-	-	-	12	56	0.035
Prostaglandin formation from dihomo γ-linoleic acid	-	-	-	-	-	-	-	-	-	-	-	-	-	-	-
Purine metabolism	-	-	-	-	-	-	-	-	-	-	-	-	-	-	-
Putative anti-inflammatory metabolites formation from EPA	-	-	-	-	-	-	-	-	-	-	-	-	-	-	-
Saturated fatty acids β-oxidation	-	-	-	-	-	-	-	-	-	-	-	-	-	-	-
Selenoamino acid metabolism	-	-	-	-	-	-	-	-	-	-	-	-	5	22	0.048
Sphingolipid metabolism	-	-	-	-	-	-	-	-	-	-	-	-	-	-	-
Starch and sucrose metabolism	-	-	-	-	-	-	-	-	-	-	-	-	-	-	-
TCA cycle	-	-	-	-	-	-	-	-	-	-	-	-	5	18	0.015
Tryptophan metabolism	-	-	-	-	-	-	-	-	-	14	63	0.013	-	-	-
Tyrosine metabolism	-	-	-	16	94	0.018	-	-	-	22	94	0.004	-	-	-
Ubiquinone biosynthesis	-	-	-	-	-	-	-	-	-	-	-	-	-	-	-
Urea cycle/amino group metabolism	-	-	-	-	-	-	6	47	0.036	11	47	0.011	-	-	-
Valine, leucine, and isoleucine degradation	-	-	-	5	26	0.036	-	-	-	-	-	-	9	31	0.004
Vitamin A metabolism	-	-	-	-	-	-	-	-	-	-	-	-	-	-	-
Vitamin B1 metabolism	-	-	-	-	-	-	2	10	0.043	-	-	-	-	-	-
Vitamin B2 metabolism	-	-	-	-	-	-	-	-	-	-	-	-	-	-	-
Vitamin B3 metabolism	-	-	-	-	-	-	-	-	-	-	-	-	-	-	-
Vitamin B5 biosynthesis	-	-	-	-	-	-	-	-	-	-	-	-	-	-	-
Vitamin B6 metabolism	-	-	-	3	8	0.010	-	-	-	4	8	0.002	-	-	-
Vitamin C metabolism	-	-	-	-	-	-	-	-	-	5	21	0.035	5	22	0.048
Vitamin D3 metabolism	-	-	-	-	-	-	2	8	0.023	-	-	-	3	11	0.045
Vitamin E metabolism	7	30	0.008	-	-	-	4	30	0.045	-	-	-	8	33	0.018
Vitamin H metabolism	-	-	-	-	-	-	-	-	-	-	-	-	-	-	-
Vitamin K metabolism	-	-	-	-	-	-	-	-	-	-	-	-	2	3	0.011

Note: Overlap size is the number of metabolic features enriched in the pathway. Pathway size is the total number of metabolic features. Only the following adducts were considered: M-H [-] , M + Cl [-] , M + ACN-H [-] , M + HCOO [-] , M(C13)–H [-] , M-H2O–H [-] , and M + Na–2H [-].

Table S2. Metabolic pathways significantly associated with single PFAS chemicals and their exposure mixture in the HILIC positive ESI column.

Pathway	HILIC ESI+ Column														
	PFHxS MWAS			PFOS MWAS			PFOA MWAS			PFNA MWAS			PFAS Mixture MWAS		
	Overlap	Pathway	p-value	Overlap	Pathway	p-value	Overlap	Pathway	p-value	Overlap	Pathway	p-value	Overlap	Pathway	p-value
Alanine and aspartate metabolism	-	-	-	-	-	-	-	-	-	-	-	-	5	23	0.019
Alkaloid biosynthesis II	-	-	-	2	6	0.026	-	-	-	-	-	-	2	6	0.037
Androgen and estrogen biosynthesis and metabolism	-	-	-	-	-	-	-	-	-	-	-	-	-	-	-
Arachidonic acid metabolism	-	-	-	14	71	0.047	-	-	-	-	-	-	-	-	-
Arginine and proline metabolism	-	-	-	-	-	-	-	-	-	-	-	-	11	34	0.001
Aspartate and asparagine metabolism	-	-	-	-	-	-	-	-	-	15	66	0.008	14	58	0.002
β-Alanine metabolism	-	-	-	-	-	-	-	-	-	-	-	-	4	14	0.009
Bile acid biosynthesis	-	-	-	-	-	-	-	-	-	-	-	-	-	-	-
Biopterin metabolism	-	-	-	-	-	-	-	-	-	5	17	0.012	5	18	0.006
Blood group biosynthesis	-	-	-	-	-	-	-	-	-	-	-	-	-	-	-
Butyrate metabolism	-	-	-	7	26	0.005	-	-	-	6	26	0.034	-	-	-
Caffeine metabolism	-	-	-	-	-	-	-	-	-	-	-	-	3	11	0.021
Carbon fixation	4	9	0.004	-	-	-	-	-	-	-	-	-	3	10	0.015
Carnitine shuttle	-	-	-	-	-	-	7	36	0.039	8	36	0.028	-	-	-
CoA catabolism	-	-	-	-	-	-	-	-	-	2	4	0.037	-	-	-
De novo fatty acid biosynthesis	13	31	0.001	12	31	0.0002	11	31	0.001	9	31	0.003	-	-	-
Di-unsaturated fatty acid β-oxidation	-	-	-	3	5	0.001	-	-	-	-	-	-	-	-	-
Drug metabolism - cytochrome P450	-	-	-	-	-	-	-	-	-	-	-	-	-	-	-
Drug metabolism - other enzymes	-	-	-	-	-	-	-	-	-	-	-	-	5	26	0.034
Dynorphin metabolism	-	-	-	-	-	-	-	-	-	-	-	-	2	6	0.037
Fatty acid activation	9	30	0.004	9	30	0.001	7	30	0.011	-	-	-	-	-	-
Fatty acid metabolism	5	17	0.015	6	17	0.001	6	17	0.002	6	17	0.003	-	-	-
Fructose and mannose metabolism	-	-	-	-	-	-	-	-	-	-	-	-	-	-	-
Glutamate metabolism	-	-	-	4	13	0.006	-	-	-	4	13	0.017	3	13	0.037
Glutathione metabolism	-	-	-	3	10	0.013	-	-	-	-	-	-	-	-	-
Glycerophospholipid metabolism	10	46	0.036	-	-	-	-	-	-	-	-	-	-	-	-
Glycine, serine, alanine, and threonine metabolism	-	-	-	13	47	0.001	11	47	0.004	12	47	0.004	9	48	0.020
Glycosphingolipid biosynthesis - ganglioseries	-	-	-	-	-	-	-	-	-	-	-	-	-	-	-
Glycosphingolipid biosynthesis - lactoseries	-	-	-	-	-	-	-	-	-	-	-	-	-	-	-
Glycosphingolipid biosynthesis - neolactoseries	-	-	-	-	-	-	-	-	-	-	-	-	-	-	-
Glycosphingolipid metabolism	-	-	-	-	-	-	-	-	-	-	-	-	-	-	-
Glyoxylate and dicarboxylate metabolism	-	-	-	-	-	-	-	-	-	-	-	-	4	9	0.002
Histidine metabolism	-	-	-	-	-	-	6	23	0.008	7	23	0.004	-	-	-
Hyaluronic acid metabolism	-	-	-	2	4	0.009	-	-	-	-	-	-	2	5	0.024
Keratan sulfate biosynthesis	-	-	-	-	-	-	-	-	-	-	-	-	-	-	-
Keratan sulfate degradation	-	-	-	3	8	0.005	-	-	-	-	-	-	-	-	-
Leukotriene metabolism	12	50	0.011	14	50	0.001	12	50	0.003	11	50	0.019	-	-	-
Linoleic acid metabolism	9	22	0.001	14	22	0.0001	-	-	-	6	22	0.012	4	21	0.048
Lysine metabolism	-	-	-	7	28	0.009	7	28	0.007	9	28	0.002	11	25	0.001
Methionine and cysteine metabolism	-	-	-	14	53	0.002	-	-	-	-	-	-	-	-	-
N-Glycan degradation	-	-	-	2	7	0.039	-	-	-	-	-	-	-	-	-

O-Glycan biosynthesis	-	-	-	-	-	-	-	-	-	-	-	-	-	-	-
Ω-3 fatty acid metabolism	5	13	0.004	4	13	0.006	-	-	-	-	-	-	-	-	-
Ω-6 fatty acid metabolism	-	-	-	4	12	0.004	5	12	0.002	-	-	-	-	-	-
Peroxisomal fatty acid oxidation	-	-	-	-	-	-	-	-	-	-	-	-	2	2	0.005
Phosphatidylinositol phosphate metabolism	7	28	0.020	-	-	-	-	-	-	-	-	-	-	-	-
Phytanic acid peroxisomal oxidation	-	-	-	4	17	0.024	-	-	-	-	-	-	-	-	-
Porphyrin metabolism	-	-	-	-	-	-	-	-	-	-	-	-	6	22	0.004
Propanoate metabolism	-	-	-	3	12	0.028	-	-	-	-	-	-	-	-	-
Prostaglandin formation from arachidonic acid	-	-	-	-	-	-	-	-	-	-	-	-	-	-	-
Prostaglandin formation from dihomo γ-linoleic acid	-	-	-	2	5	0.016	-	-	-	-	-	-	-	-	-
Purine metabolism	14	58	0.008	12	58	0.030	-	-	-	-	-	-	-	-	-
Putative anti-inflammatory metabolites formation from EPA	-	-	-	7	21	0.001	-	-	-	-	-	-	-	-	-
Saturated fatty acids β-oxidation	-	-	-	5	17	0.005	-	-	-	-	-	-	-	-	-
Selenoamino acid metabolism	-	-	-	6	22	0.006	-	-	-	-	-	-	4	21	0.048
Sphingolipid metabolism	-	-	-	-	-	-	2	2	0.007	-	-	-	-	-	-
Starch and sucrose metabolism	-	-	-	5	19	0.010	-	-	-	-	-	-	-	-	-
TCA cycle	-	-	-	4	18	0.032	-	-	-	-	-	-	5	21	0.012
Tryptophan metabolism	-	-	-	-	-	-	-	-	-	-	-	-	-	-	-
Tyrosine metabolism	-	-	-	-	-	-	-	-	-	-	-	-	22	94	0.001
Ubiquinone biosynthesis	-	-	-	2	7	0.039	-	-	-	-	-	-	-	-	-
Urea cycle/amino group metabolism	-	-	-	-	-	-	-	-	-	-	-	-	13	47	0.001
Valine, leucine, and isoleucine degradation	-	-	-	7	31	0.018	-	-	-	7	31	0.031	-	-	-
Vitamin A metabolism	9	38	0.020	11	38	0.001	-	-	-	-	-	-	-	-	-
Vitamin B1 metabolism	-	-	-	-	-	-	-	-	-	-	-	-	-	-	-
Vitamin B2 metabolism	-	-	-	2	4	0.009	2	4	0.030	-	-	-	-	-	-
Vitamin B3 metabolism	8	23	0.002	5	23	0.030	-	-	-	-	-	-	-	-	-
Vitamin B5 biosynthesis	-	-	-	-	-	-	-	-	-	3	10	0.042	-	-	-
Vitamin B6 metabolism	-	-	-	-	-	-	-	-	-	-	-	-	3	8	0.008
Vitamin C metabolism	-	-	-	5	22	0.023	-	-	-	-	-	-	6	21	0.004
Vitamin D3 metabolism	-	-	-	-	-	-	3	11	0.042	-	-	-	-	-	-
Vitamin E metabolism	8	33	0.020	9	33	0.003	7	33	0.021	-	-	-	6	30	0.022
Vitamin H metabolism	2	4	0.042	2	4	0.009	2	4	0.030	2	4	0.037	-	-	-
Vitamin K metabolism	2	3	0.021	-	-	-	-	-	-	-	-	-	-	-	-

Note: Overlap size is the number of metabolic features enriched in the pathway. Pathway size is the total number of metabolic features. Only the following adducts were considered: $M^{[1+]}$, $M + H^{[1+]}$, $M\text{-}H2O + H^{[1+]}$, $M + Na^{[1+]}$, $M + K^{[1+]}$, $M\text{+}2H^{[2+]}$, and $M(C13)\text{+ }2H^{[2+]}$.
